# 2-Chloro-6-(2,3-di­chloro­benzene­sulfonamido)­benzoic acid

**DOI:** 10.1107/S1600536813011574

**Published:** 2013-05-04

**Authors:** Ayesha Munir, Hafiz Mubashar-ur-Rehman, Abdullah M. Asiri, Islam Ullah Khan, Muhammad Nadeem Arshad

**Affiliations:** a Department of Chemistry, Materials Chemistry Laboratory, GC University, Lahore 54000, Pakistan; b Chemistry Department, Faculty of Science, King Abdulaziz University, PO Box 80203, Jeddah 21589, Saudi Arabia; c Center of Excellence for Advanced Materials Research (CEAMR), King Abdulaziz University, PO Box 80203, Jeddah 21589, Saudi Arabia

## Abstract

In the title compound, C_13_H_8_Cl_3_NO_4_S, the aromatic rings are oriented at a dihedral angle of 68.94 (1)° and the mol­ecule adopts a V-shape. An intra­molecular N—H⋯O inter­action generates a six-membered *S*(6) ring motif. In the crystal, pairs of O—H⋯O hydrogen bonds involving the carb­oxy group link the mol­ecules into inversion dimers with an *R*_2_^2^(8) motif. N—H⋯O and non-classical C—H⋯O inter­actions connect the mol­ecules, forming sheets propagating in (100).

## Related literature

For the synthesis, see: Arshad *et al.* (2012 ▶) For related structures, see: Arshad *et al.* (2009 ▶, 2011 ▶). For graph-set notation, see: Bernstein *et al.* (1995 ▶).
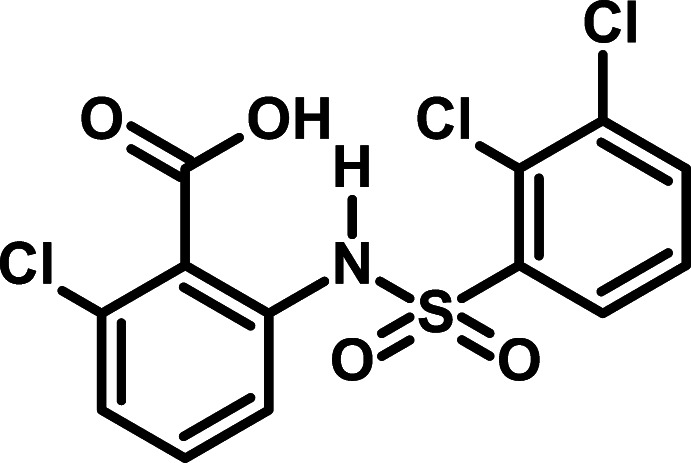


## Experimental

### 

#### Crystal data

C_13_H_8_Cl_3_NO_4_S
*M* *_r_* = 380.61
Monoclinic, 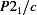

*a* = 9.0164 (3) Å
*b* = 18.6017 (5) Å
*c* = 9.8574 (3) Å
β = 111.653 (3)°
*V* = 1536.62 (8) Å^3^
*Z* = 4
Cu *K*α radiation
μ = 6.83 mm^−1^
*T* = 296 K
0.38 × 0.20 × 0.18 mm

#### Data collection

Agilent SuperNova (Dual, Cu at zero, Atlas, CCD) diffractometer
Absorption correction: multi-scan (*CrysAlis PRO*; Agilent, 2012 ▶) *T*_min_ = 0.467, *T*_max_ = 1.000
11742 measured reflections
3018 independent reflections
2597 reflections with *I* > 2σ(*I*)
*R*_int_ = 0.025

#### Refinement

*R*[*F* ^2^ > 2σ(*F* ^2^)] = 0.038
*wR*(*F* ^2^) = 0.104
*S* = 1.04
3018 reflections
201 parameters
H-atom parameters constrained
Δρ_max_ = 0.55 e Å^−3^
Δρ_min_ = −0.60 e Å^−3^

### 

Data collection: *CrysAlis PRO* (Agilent, 2012 ▶); cell refinement: *CrysAlis PRO*; data reduction: *CrysAlis PRO*; program(s) used to solve structure: *SHELXS97* (Sheldrick, 2008 ▶); program(s) used to refine structure: *SHELXL97* (Sheldrick, 2008 ▶); molecular graphics: *PLATON* (Spek, 2009 ▶); software used to prepare material for publication: *WinGX* (Farrugia, 2012 ▶) and *X-SEED* (Barbour, 2001 ▶).

## Supplementary Material

Crystal structure: contains datablock(s) I, global. DOI: 10.1107/S1600536813011574/hg5312sup1.cif

Structure factors: contains datablock(s) I. DOI: 10.1107/S1600536813011574/hg5312Isup2.hkl

Supplementary material file. DOI: 10.1107/S1600536813011574/hg5312Isup3.cml

Additional supplementary materials:  crystallographic information; 3D view; checkCIF report

## Figures and Tables

**Table 1:** Hydrogen-bond geometry (Å, °)

*D*—H⋯*A*	*D*—H	H⋯*A*	*D*⋯*A*	*D*—H⋯*A*
N1—H1⋯O3	0.86	2.55	2.940 (2)	108
C12—H12⋯O3^i^	0.93	2.59	3.425 (3)	150
O3—H3⋯O4^ii^	0.82	1.85	2.666 (2)	176
N1—H1⋯O2^iii^	0.86	2.30	3.128 (2)	162
C5—H5⋯O4^iv^	0.93	2.53	3.256 (4)	135
C10—H10⋯O1^v^	0.93	2.51	3.165 (3)	127

Symmetry codes: (i) 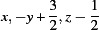; (ii) 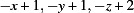; (iii) 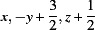; (iv) 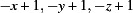; (v) 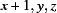.

**Fig. 1.**
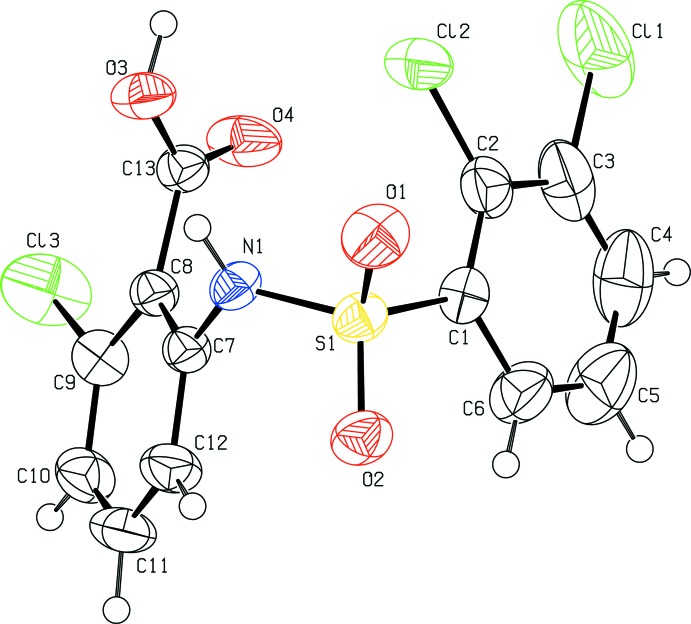
The labelled structure of (C13 H8 Cl3 N O4 S) with 50% probability of thermal ellipsoids.

**Fig. 2.**
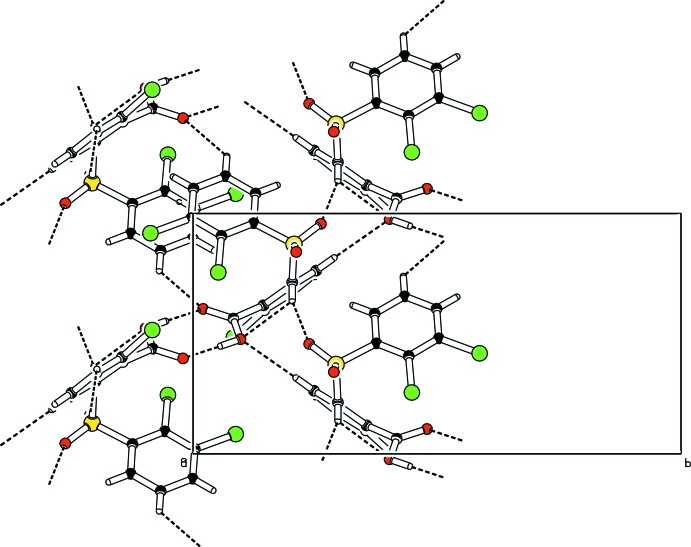
A perspective view showing two dimensional network generating through O—H···O, N—H···O and C—H···O hydrogen bonds, drawn using dashed lines.

## supplementary crystallographic information

### Comment

In connection to synthesis of halogenated sulfonamide derivatives 2-Chloro-4-(2-iodobenzenesulfonamido)benzoic acid (Arshad *et al.*, 2011) and 2-Chloro-5-(2-iodobenzenesulfonamido)benzoic acid (Arshad *et al.*, 2009), we are reporting the crystal structure of title compound.

The two aromatic rings [(C1—C6) & (C7—C12)] in the structure of molecule are oriented at dihedral angle of 68.94 (1)°. The carboxylic group (C13/O3/O4) is twisted at 58.11 (1)° with respect to its mother aromatic ring (C7—C12) and its atoms C13, O3 & O4 are away by -0.1938 (35)Å , 0.6924 (39)Å , -1.1739 (39)Å repectively from the mean plane generating from atoms C7/C8/C9/C10/C11/C12 with the r.m.s deviation of 0.0183 (15) Å. The amino and carboxylic groups are involved in classical N1—H1···O3 intramolecular hydrogen bonding interaction and produce six membered ring motif *S*^1^_1_ (6) (Bernstein *et al.* 1995) which is oriented at dihedral angles of 54.30 (11)° & 18.98 (12)° with respect to two aromatic rings [(C1—C6) & (C7—C12)], respectively. On the other hand the amino group get connected with oxygen of SO_2_ to form intermolecular N1—H1···O2 hydrogen bond. The carboxylic group gives typical inversion dimerization by generating eight membered ring motif *R*^2^_2_ (8) (Bernstein *et al.* 1995) through O3—H3···O4 interaction. The non-clasical C—H···O type interaction have also been observed in the molecule (Fig. 2) for which symmetry detail are available in Table 1.

### Experimental

The title compound was synthesised following the literature method (Arshad *et al.*, 2012) and recrystallized from ethylacetate under slow evaporation at room temperature.

### Refinement

All the H-atoms were positioned with idealized geometry with C—H = 0.93 Å, N—H = 0.86 Å, O—H = 0.82 Å and were refined as riding with *U*_iso_(H) = 1.2 *U*_eq_(K), where K = C, N & O for all H-atoms. The reflections (0 1 2), (1 3 1), (1 0 0), (2 1 0) & (0 2 0) are omitted in final refinement.

### Crystal data

**Table:**

C_13_H_8_Cl_3_NO_4_S	*F*(000) = 768
*M**_r_* = 380.61	*D*_x_ = 1.645 Mg m^−^^3^
Monoclinic, *P*2_1_/*c*	Cu *K*α radiation, λ = 1.54184 Å
Hall symbol: -P 2ybc	Cell parameters from 5339 reflections
*a* = 9.0164 (3) Å	θ = 4.8–73.0°
*b* = 18.6017 (5) Å	µ = 6.83 mm^−^^1^
*c* = 9.8574 (3) Å	*T* = 296 K
β = 111.653 (3)°	Prismatic, colorless
*V* = 1536.62 (8) Å^3^	0.38 × 0.20 × 0.18 mm
*Z* = 4	

### Data collection

**Table:**

Agilent SuperNova (Dual, Cu at zero, Atlas, CCD) diffractometer	3018 independent reflections
Radiation source: SuperNova (Cu) X-ray Source	2597 reflections with *I* > 2σ(*I*)
Mirror monochromator	*R*_int_ = 0.025
ω scans	θ_max_ = 73.1°, θ_min_ = 5.4°
Absorption correction: multi-scan (*CrysAlis PRO*; Agilent, 2012)	*h* = −10→11
*T*_min_ = 0.467, *T*_max_ = 1.000	*k* = −22→22
11742 measured reflections	*l* = −12→11

### Refinement

**Table:**

Refinement on *F*^2^	Secondary atom site location: difference Fourier map
Least-squares matrix: full	Hydrogen site location: inferred from neighbouring sites
*R*[*F*^2^ > 2σ(*F*^2^)] = 0.038	H-atom parameters constrained
*wR*(*F*^2^) = 0.104	*w* = 1/[σ^2^(*F*_o_^2^) + (0.0471*P*)^2^ + 1.0131*P*] where *P* = (*F*_o_^2^ + 2*F*_c_^2^)/3
*S* = 1.04	(Δ/σ)_max_ < 0.001
3018 reflections	Δρ_max_ = 0.55 e Å^−^^3^
201 parameters	Δρ_min_ = −0.60 e Å^−^^3^
0 restraints	Extinction correction: *SHELXL97* (Sheldrick, 2008), Fc^*^=kFc[1+0.001xFc^2^λ^3^/sin(2θ)]^-1/4^
Primary atom site location: structure-invariant direct methods	Extinction coefficient: 0.0022 (2)

### Special details

**Table:**

Geometry. All esds (except the esd in the dihedral angle between two l.s. planes) are estimated using the full covariance matrix. The cell esds are taken into account individually in the estimation of esds in distances, angles and torsion angles; correlations between esds in cell parameters are only used when they are defined by crystal symmetry. An approximate (isotropic) treatment of cell esds is used for estimating esds involving l.s. planes.
Refinement. Refinement of F^2^ against ALL reflections. The weighted R-factor wR and goodness of fit S are based on F^2^, conventional R-factors R are based on F, with F set to zero for negative F^2^. The threshold expression of F^2^ > 2sigma(F^2^) is used only for calculating R-factors(gt) etc. and is not relevant to the choice of reflections for refinement. R-factors based on F^2^ are statistically about twice as large as those based on F, and R- factors based on ALL data will be even larger.

### Fractional atomic coordinates and isotropic or equivalent isotropic displacement parameters (Å^2^)

**Table:**

	*x*	*y*	*z*	*U*_iso_*/*U*_eq_	
Cl1	0.21203 (15)	0.41272 (5)	0.58195 (14)	0.1161 (5)	
Cl2	0.17428 (9)	0.55156 (5)	0.74492 (8)	0.0718 (3)	
Cl3	0.94473 (9)	0.58369 (5)	1.01517 (11)	0.0797 (3)	
S1	0.24536 (6)	0.70690 (3)	0.62789 (6)	0.03763 (17)	
O1	0.1045 (2)	0.71147 (11)	0.6596 (2)	0.0562 (5)	
O2	0.2792 (2)	0.76184 (9)	0.54279 (18)	0.0482 (4)	
O3	0.5137 (2)	0.59728 (8)	1.02304 (19)	0.0460 (4)	
H3	0.4816	0.5600	1.0473	0.055*	
O4	0.6012 (2)	0.52066 (9)	0.8971 (2)	0.0572 (5)	
N1	0.3928 (2)	0.70318 (10)	0.78616 (19)	0.0357 (4)	
H1	0.3706	0.7032	0.8638	0.043*	
C1	0.2524 (3)	0.62421 (13)	0.5405 (2)	0.0417 (5)	
C2	0.2255 (3)	0.55805 (14)	0.5932 (3)	0.0510 (6)	
C3	0.2403 (4)	0.49629 (16)	0.5195 (4)	0.0693 (9)	
C4	0.2779 (4)	0.5009 (2)	0.3962 (4)	0.0843 (11)	
H4	0.2867	0.4592	0.3476	0.101*	
C5	0.3022 (5)	0.5663 (2)	0.3450 (4)	0.0804 (10)	
H5	0.3269	0.5691	0.2615	0.096*	
C6	0.2903 (4)	0.62797 (17)	0.4168 (3)	0.0583 (7)	
H6	0.3078	0.6724	0.3823	0.070*	
C7	0.5563 (2)	0.69989 (11)	0.8019 (2)	0.0327 (4)	
C8	0.6539 (2)	0.64427 (11)	0.8821 (2)	0.0329 (4)	
C9	0.8156 (3)	0.64584 (13)	0.9024 (3)	0.0418 (5)	
C10	0.8775 (3)	0.69735 (14)	0.8382 (3)	0.0485 (6)	
H10	0.9852	0.6970	0.8518	0.058*	
C11	0.7777 (3)	0.74919 (15)	0.7535 (3)	0.0536 (7)	
H11	0.8175	0.7829	0.7061	0.064*	
C12	0.6188 (3)	0.75223 (13)	0.7376 (3)	0.0467 (6)	
H12	0.5540	0.7892	0.6840	0.056*	
C13	0.5870 (3)	0.58240 (11)	0.9374 (2)	0.0348 (4)	

### Atomic displacement parameters (Å^2^)

**Table:**

	*U*^11^	*U*^22^	*U*^33^	*U*^12^	*U*^13^	*U*^23^
Cl1	0.1280 (9)	0.0446 (5)	0.1266 (9)	−0.0192 (5)	−0.0106 (7)	0.0082 (5)
Cl2	0.0734 (5)	0.0782 (5)	0.0597 (4)	−0.0271 (4)	0.0197 (3)	0.0177 (4)
Cl3	0.0502 (4)	0.0720 (5)	0.1115 (7)	0.0207 (3)	0.0236 (4)	0.0361 (5)
S1	0.0363 (3)	0.0420 (3)	0.0365 (3)	0.0046 (2)	0.0158 (2)	0.0039 (2)
O1	0.0388 (9)	0.0760 (13)	0.0587 (11)	0.0094 (8)	0.0238 (8)	0.0034 (9)
O2	0.0531 (9)	0.0456 (10)	0.0438 (9)	0.0048 (7)	0.0153 (7)	0.0123 (7)
O3	0.0692 (11)	0.0324 (8)	0.0514 (10)	−0.0026 (8)	0.0397 (9)	0.0004 (7)
O4	0.0914 (14)	0.0287 (8)	0.0748 (13)	−0.0047 (8)	0.0580 (11)	−0.0055 (8)
N1	0.0380 (9)	0.0422 (10)	0.0303 (9)	0.0018 (7)	0.0167 (7)	0.0005 (7)
C1	0.0419 (11)	0.0449 (13)	0.0356 (11)	−0.0006 (10)	0.0111 (9)	−0.0023 (9)
C2	0.0446 (13)	0.0496 (15)	0.0468 (14)	−0.0081 (11)	0.0027 (11)	0.0017 (11)
C3	0.0639 (17)	0.0444 (16)	0.072 (2)	−0.0044 (13)	−0.0075 (15)	−0.0037 (14)
C4	0.095 (2)	0.070 (2)	0.070 (2)	0.0129 (19)	0.0098 (19)	−0.0296 (18)
C5	0.108 (3)	0.078 (2)	0.0594 (19)	0.012 (2)	0.0362 (19)	−0.0156 (17)
C6	0.0753 (18)	0.0590 (17)	0.0460 (14)	0.0016 (14)	0.0285 (13)	−0.0020 (12)
C7	0.0369 (10)	0.0302 (10)	0.0325 (10)	−0.0020 (8)	0.0148 (8)	−0.0012 (8)
C8	0.0405 (10)	0.0270 (10)	0.0336 (10)	−0.0026 (8)	0.0166 (8)	−0.0021 (8)
C9	0.0396 (11)	0.0391 (12)	0.0474 (13)	0.0024 (9)	0.0168 (10)	0.0002 (10)
C10	0.0369 (11)	0.0556 (15)	0.0568 (15)	−0.0089 (10)	0.0218 (11)	−0.0030 (12)
C11	0.0518 (14)	0.0530 (16)	0.0602 (16)	−0.0145 (11)	0.0257 (12)	0.0121 (12)
C12	0.0468 (13)	0.0401 (13)	0.0515 (14)	−0.0042 (10)	0.0161 (11)	0.0126 (10)
C13	0.0418 (11)	0.0297 (10)	0.0357 (11)	0.0013 (8)	0.0174 (9)	0.0012 (8)

### Geometric parameters (Å, º)

**Table:**

Cl1—C3	1.725 (3)	C4—C5	1.366 (5)
Cl2—C2	1.725 (3)	C4—H4	0.9300
Cl3—C9	1.724 (2)	C5—C6	1.373 (4)
S1—O1	1.4179 (17)	C5—H5	0.9300
S1—O2	1.4247 (17)	C6—H6	0.9300
S1—N1	1.6357 (18)	C7—C12	1.389 (3)
S1—C1	1.776 (2)	C7—C8	1.399 (3)
O3—C13	1.279 (3)	C8—C9	1.397 (3)
O3—H3	0.8200	C8—C13	1.493 (3)
O4—C13	1.238 (3)	C9—C10	1.375 (3)
N1—C7	1.426 (3)	C10—C11	1.372 (4)
N1—H1	0.8600	C10—H10	0.9300
C1—C6	1.384 (3)	C11—C12	1.383 (3)
C1—C2	1.391 (3)	C11—H11	0.9300
C2—C3	1.391 (4)	C12—H12	0.9300
C3—C4	1.380 (5)		
			
O1—S1—O2	119.37 (11)	C5—C6—C1	120.3 (3)
O1—S1—N1	105.73 (10)	C5—C6—H6	119.9
O2—S1—N1	108.47 (10)	C1—C6—H6	119.9
O1—S1—C1	110.74 (12)	C12—C7—C8	120.0 (2)
O2—S1—C1	106.38 (11)	C12—C7—N1	119.82 (19)
N1—S1—C1	105.32 (10)	C8—C7—N1	120.19 (18)
C13—O3—H3	109.5	C9—C8—C7	118.12 (19)
C7—N1—S1	123.33 (14)	C9—C8—C13	120.35 (19)
C7—N1—H1	118.3	C7—C8—C13	121.44 (18)
S1—N1—H1	118.3	C10—C9—C8	121.9 (2)
C6—C1—C2	120.5 (2)	C10—C9—Cl3	118.17 (18)
C6—C1—S1	116.6 (2)	C8—C9—Cl3	119.94 (18)
C2—C1—S1	122.88 (19)	C11—C10—C9	118.9 (2)
C1—C2—C3	118.2 (3)	C11—C10—H10	120.5
C1—C2—Cl2	121.7 (2)	C9—C10—H10	120.5
C3—C2—Cl2	120.2 (2)	C10—C11—C12	121.2 (2)
C4—C3—C2	120.7 (3)	C10—C11—H11	119.4
C4—C3—Cl1	119.1 (3)	C12—C11—H11	119.4
C2—C3—Cl1	120.2 (3)	C11—C12—C7	119.8 (2)
C5—C4—C3	120.4 (3)	C11—C12—H12	120.1
C5—C4—H4	119.8	C7—C12—H12	120.1
C3—C4—H4	119.8	O4—C13—O3	123.6 (2)
C4—C5—C6	120.0 (3)	O4—C13—C8	119.58 (19)
C4—C5—H5	120.0	O3—C13—C8	116.82 (18)
C6—C5—H5	120.0		
			
O1—S1—N1—C7	−178.47 (17)	S1—C1—C6—C5	178.3 (3)
O2—S1—N1—C7	−49.3 (2)	S1—N1—C7—C12	55.1 (3)
C1—S1—N1—C7	64.24 (19)	S1—N1—C7—C8	−125.01 (19)
O1—S1—C1—C6	132.5 (2)	C12—C7—C8—C9	3.9 (3)
O2—S1—C1—C6	1.4 (2)	N1—C7—C8—C9	−175.94 (19)
N1—S1—C1—C6	−113.6 (2)	C12—C7—C8—C13	−172.5 (2)
O1—S1—C1—C2	−49.0 (2)	N1—C7—C8—C13	7.6 (3)
O2—S1—C1—C2	179.83 (19)	C7—C8—C9—C10	−4.4 (3)
N1—S1—C1—C2	64.8 (2)	C13—C8—C9—C10	172.1 (2)
C6—C1—C2—C3	1.0 (4)	C7—C8—C9—Cl3	173.60 (17)
S1—C1—C2—C3	−177.45 (19)	C13—C8—C9—Cl3	−9.9 (3)
C6—C1—C2—Cl2	−178.7 (2)	C8—C9—C10—C11	1.0 (4)
S1—C1—C2—Cl2	2.9 (3)	Cl3—C9—C10—C11	−177.0 (2)
C1—C2—C3—C4	−1.1 (4)	C9—C10—C11—C12	3.0 (4)
Cl2—C2—C3—C4	178.6 (2)	C10—C11—C12—C7	−3.4 (4)
C1—C2—C3—Cl1	178.77 (19)	C8—C7—C12—C11	−0.2 (4)
Cl2—C2—C3—Cl1	−1.5 (3)	N1—C7—C12—C11	179.7 (2)
C2—C3—C4—C5	0.4 (5)	C9—C8—C13—O4	−56.9 (3)
Cl1—C3—C4—C5	−179.4 (3)	C7—C8—C13—O4	119.5 (2)
C3—C4—C5—C6	0.4 (6)	C9—C8—C13—O3	124.7 (2)
C4—C5—C6—C1	−0.5 (5)	C7—C8—C13—O3	−58.9 (3)
C2—C1—C6—C5	−0.2 (4)		

### Hydrogen-bond geometry (Å, º)

**Table:**

*D*—H···*A*	*D*—H	H···*A*	*D*···*A*	*D*—H···*A*
N1—H1···O3	0.86	2.55	2.940 (2)	108
C12—H12···O3^i^	0.93	2.59	3.425 (3)	150
O3—H3···O4^ii^	0.82	1.85	2.666 (2)	176
N1—H1···O2^iii^	0.86	2.30	3.128 (2)	162
C5—H5···O4^iv^	0.93	2.53	3.256 (4)	135
C10—H10···O1^v^	0.93	2.51	3.165 (3)	127

Symmetry codes: (i) *x*, −*y*+3/2, *z*−1/2; (ii) −*x*+1, −*y*+1, −*z*+2; (iii) *x*, −*y*+3/2, *z*+1/2; (iv) −*x*+1, −*y*+1, −*z*+1; (v) *x*+1, *y*, *z*.
